# Profile of substance use disorders in elderly psychiatric inpatients

**DOI:** 10.1192/j.eurpsy.2023.1986

**Published:** 2023-07-19

**Authors:** M. Karoui, R. Kammoun, H. Nefzi, F. Ellouze

**Affiliations:** 1 Psychiatrie, Faculté de médecine de Tunis, Tunis, Tunisia

## Abstract

**Introduction:**

Substance abuse is a common problem associated with significant morbidity and mortality, but is often underdiagnosed and unrecognized in geriatric patient populations.

**Objectives:**

to determine the prevalence of substance use disorders geriatricic inpatient population.

**Methods:**

Data from 2010 to 2020 were retrospectively reviewed from a clinical database. 148 admissions of patients older than 60 years were identified. Descriptive statistics were used to group patients with and without a diagnosis of substance use, which included intoxication, withdrawal, abuse, dependence, and substance-induced disorders.

**Results:**

There were 148 hospital admissions for patients over 60 years of age, with a mean age of 72.38 ± 5.64 years and a mean length of stay of 13.91 ± 14.15 days. Of all admissions, 44% (n=64) were associated with at least one substance use diagnosis. In this group of 64 patients, the most frequently used substance was tobacco with associated disorders ( 65% N=42). The prevalence of other substance use diagnoses was as follows: sedative-hypnotic abuse/dependence 32% (N=21), cannabis abuse 10% (N=6), alcohol-related disorder 12.5% (N=8). Compared with patients without a substance abuse diagnosis, these patients were significantly younger, had shorter lengths of stay, were less likely to be readmitted, and were more likely to be single men

**Conclusions:**

Given the inherent difficulties in diagnosing substance use disorders and the retrospective nature of this study, the true prevalence of substance use disorders in elderly psychiatric inpatients is likely higher than found. Cross-sectional or cohort studies are more appropriate to shed light on this condition.

**Disclosure of Interest:**

None Declared

